# Explaining variance in health literacy among people with type 2 diabetes: the association between health literacy and health behaviour and empowerment

**DOI:** 10.1186/s12889-020-8274-z

**Published:** 2020-02-03

**Authors:** Hanne Søberg Finbråten, Øystein Guttersrud, Gun Nordström, Kjell Sverre Pettersen, Anne Trollvik, Bodil Wilde-Larsson

**Affiliations:** 1 Department of Health and Nursing Sciences, Faculty of Social and Health Sciences, Inland Norway University of Applied Sciences, PO Box 400, N-2418 Elverum, Norway; 2 Department of Health Sciences, Faculty of Health, Science and Technology, Nursing science, Karlstad University, SE-65188 Karlstad, Sweden; 3 Norwegian Centre for Science Education, Faculty of Mathematics and Natural Sciences, University of Oslo, PO Box 1106, Blindern, N-0317 Oslo, Norway; 4 Department of Nursing and Health Promotion, Faculty of Health Sciences, OsloMet – Oslo Metropolitan University, PO Box 4, St Olavs plass, N-0130 Oslo, Norway

**Keywords:** Empowerment, General health, Health behaviour, Health literacy, HLS-Q12, Sequential multiple regression analysis, Type 2 diabetes

## Abstract

**Background:**

To reflect the health literacy (HL) skills needed for managing type 2 diabetes (T2DM) in everyday life, HL in people with T2DM should be measured from a broader perspective than basic skills, such as proficiency in reading and writing. The HLS-Q12, based on the European Health Literacy Survey Questionnaire (HLS-EU-Q47), assesses four cognitive domains across three health domains. International studies on people with T2DM show inconsistent results regarding the association between HL and general health and the association between HL and glycaemic control. Moreover, knowledge is needed related to the link between HL and empowerment for those with T2DM. The aims of this study were to examine the association between i) HL and general health and diabetes outcomes, ii) HL and health behaviours and iii) HL and empowerment in people with T2DM.

**Methods:**

During March and April 2015, 388 adults with T2DM responded to a paper-and-pencil self-administered questionnaire. A sequential multiple regression analysis was applied to explore the association between HL, as measured by the HLS-Q12, and health conditions, HbA1c, health behaviours and empowerment.

**Results:**

For people with T2DM, higher levels of HL were associated with higher levels of education, better overall health conditions and higher self-perceived empowerment. No empirical evidence strengthening either the link between HL and glycaemic control or the link between HL and health behaviours was found.

**Conclusions:**

The independent variables education level, overall health condition and empowerment explained about one-third of the total observed variance in HL.

## Background

Health literacy (HL) is an essential individual competence necessary for dealing with health information provided, making appropriate health-related decisions and managing health in different situations [1]. Sørensen et al. [2] defined HL as follows: *Health literacy is linked to literacy and entails people’s knowledge, motivation and competence to access, understand, appraise, and apply health information in order to make judgments and take decisions in everyday life concerning healthcare, disease prevention and health promotion to maintain or improve quality of life during the life course* (p. 3)*.* Having sufficient HL might be more important than ever before because people are expected to participate in health decisions and to take responsibility for their own health despite more complicated health problems and the need to navigate a more complex health system [3].

Studies have linked poor diabetes knowledge [4–6], poor glycaemic control [5, 7–9] and diabetic retinopathy [7] in people with type 2 diabetes (T2DM) to low HL. Using a modified version of the Functional, Communicative and Critical Health Literacy scale (FCCHL) [10], Finbråten et al. [11] found that lower HL is linked with poorer self-reported general health in people with T2DM. Similarly, Al Sayah, Qiu and Johnson [12] found that low HL is associated with lower health-related ‘quality of life’ measures in people with T2DM. However, neither the links between low HL and diabetes outcomes, such as poor glycaemic control [13–15], nor the links between HL and other self-reported health-related measures, such as general health and quality of life, are consistent [13, 16, 17]. Based on their systematic review of reviews, Caruso et al. [18] also revealed gaps in knowledge about the relations between HL and health outcomes. Thus, further studies are needed.

The prevalence of T2DM is increasing worldwide, and T2DM is strongly associated with lifestyle and health behaviours [19]. Physical activity has a beneficial effect on blood glucose levels, whereas physical inactivity, the use of tobacco and high alcohol consumption could increase the risk for complications, such as cardiovascular diseases [19, 20]. Despite T2DM having been associated with health behaviours, such as low physical activity [19], few studies have investigated the association between HL and health behaviours in people with T2DM. Some studies have linked low HL to physical inactivity, but these studies did not differentiate between type 1 and type 2 diabetes [4, 21]. The associations between HL and smoking behaviours and alcohol consumption are not strongly supported [22–26].

HL may facilitate the self-management of T2DM [27], active participation in diabetes treatment [28] and interactive communication with health professionals. ‘Empowerment’—individuals’ ability to make decisions and to maintain control over their personal lives [29]—refers to self-management and control over one’s own health. Empowerment has become a major goal in health communication [30] and is also viewed as a goal of HL itself [31]. Theoretical relationships have been established between HL and empowerment [1, 2, 32], where high HL is believed to increase opportunities for autonomy and empowerment in health-related decision-making [32, 33]. Wang et al. [27] emphasised the importance of HL and empowerment for self-management in people with T2DM. However, the association between HL and empowerment in people with T2DM has not yet been fully explored.

Research on the links between HL and health outcomes in general and between HL and health outcomes related to T2DM specifically have measured HL as a set of basic skills in clinical settings, such as numeracy and reading, which one might refer to as ‘functional’ HL. Examples of such measurement scales are the Test of Functional Health Literacy in Adults (TOFHLA) [34] (or the S-TOFHLA [35]), the Rapid Estimate of Adult Literacy in Medicine (REALM) [36] and Chew’s ‘brief questions to identify patients with inadequate HL’ [37]. However, people with T2DM require proficiencies that surpass basic skills if they are to utilise health information in their everyday management of the disease. The relationship between HL and health outcomes in general and between health outcomes of T2DM specifically should be further explored using instruments that capture the broader aspects of HL, such as the capacity to find, understand, evaluate and apply health information across different contexts; health care, disease prevention and health promotion. These capacities are related to the definition of HL used in this article. The European Health Literacy Survey Questionnaire (HLS-EU-Q47) was developed on the basis of this framework [2, 38, 39]. However, Finbråten et al. [40] and Huang et al. [41] have revealed several psychometric weaknesses of the HLS-EU-Q47 when validated in people with T2DM and in people with stroke, respectively. The newly developed and shortened version of this instrument, the HLS-Q12, which has enhanced psychometric properties [42, 43], was well-suited for HL screenings in the study sample of people with T2DM.

Against this background, the aims of this study were to examine the association between i) HL and general health and diabetes outcomes, ii) HL and health behaviours and iii) HL and empowerment in people with T2DM.

## Methods

### Study design

This cross-sectional study was conducted based on data from adults with T2DM recruited from the Norwegian Diabetes Association (NDA)—a member of the International Diabetes Federation. NDA is an independent organisation that aims to serve people with diabetes, and membership is voluntary.

### Participants and data collection

According to the Norwegian Prescription Database 149,057 people were pharmacologically treated for T2DM in 2014 (87% of these had an age of 50 or older and 43% were females) [44]. At the time of sampling, 16,754 people with T2DM (average age 68 years and 53% females) were members of the NDA (Norwegian Diabetes Association, personal communication, 7 May 2015). After careful instructions from the researchers, the NDA drew a random sample of 999 people with T2DM from the 7655 members residing in nine of Norway’s 19 counties (members from the other counties participated in a pilot study that was conducted 1 year prior). All parts of the country were represented in the current study. An inclusion criteria was being diagnosed with T2DM. Further, the NDA was asked to draw a random sample from the member list, but make sure that the gender and age composition of the sample matched the distribution of gender and age of the members in the selected counties. Assuming 80% power, a small to medium effect size and having six predictors, a sample size of 142 would be sufficient for regression analysis. When using a sample size calculator and considering a confidence level of 95% and a confidence interval of 5, a sample of 376 is recommended. Thus, invitation was sent to 999 people, expecting that about one third should return a completed questionnaire.

Using a self-administrated paper-and-pencil questionnaire, the data were collected between March and April 2015. As the questionnaire was distributed via regular mail, two questionnaires were returned due to unknown addresses. Responses were received from 406 individuals. Eighteen respondents who reported type 1 diabetes were excluded as they did not meet the inclusion criteria. Furthermore, 31 individuals reported health conditions that were incompatible with responding to the questionnaire and were consequently excluded. Hence, the analyses were based on responses from 388 individuals (response rate 41% [388/948]).

In our sample, just over half of the 388 respondents were men (Table 1). The average age was 73 years. Just below one-third had compulsory comprehensive school as their highest completed education, and about the same fraction had completed education at a university or university college level. The majority of the respondents reported fairly good to very good general health.

**Table 1:** Sample characteristics (*n* = 388)

Characteristic	
Gender	*n* (%)
Male	207 (53)
Female	165 (43)
Missing	14 (4)
Education	*n* (%)
Compulsory comprehensive school	111 (29)
Upper secondary school	84 (22)
University/university college	118 (30)
Other	51(13)
Missing	22 (6)
General health status	*n* (%)
Very good	19 (5)
Good	150 (39)
Fairly good	146 (38)
Bad	57 (15)
Very bad	7 (2)
Missing	7 (2)
Age	
Mean (*sd*)	73 (8.6)
Median	74
Lowest-highest	50 − 92
Missing	13
HbA1c (%)^a^	
Mean (*sd*)	7.29 (0.98)
Lowest-highest	5.4–13.0
Missing	76 (20)

*Note:* Continuous data: mean (standard deviation [sd]); categorical data: frequencies (percentage [%])

^a^Highest recommended HbA1c level in people with T2DM is 7% (53 mmol/mol)

### Measures

HL was measured using the HLS-Q12 instrument developed by Finbråten et al. [42]. The respondents were asked to report demographic variables (gender, age and education level), health behaviours (physical activity, alcohol consumption and tobacco use), general health condition (‘How is your health in general?’), items intended to be indicators of empowerment and their latest measured glycated haemoglobin (HbA1c) level. In Norway, HbA1c is measured at least twice a year in people with T2DM [45]. They were also asked whether they had communicated with a registered nurse about health-related issues during the past 12 months.

#### Health literacy

Using the 12-item HLS-Q12 [42], which has been validated in the Norwegian population [42, 43] and for Norwegians with T2DM (see below) [43], HL was measured across the four cognitive domains (access, understand, appraise and apply health information) and three health domains (health care, disease prevention and health promotion) of the model developed by Sørensen et al. [2]. The 12-item HLS-Q12 scale, for which each cell of this 4 × 3 matrix of HL is represented by one item, has a 4-point rating scale; very difficult (1), difficult (2), easy (3) and very easy (4), where the higher score sums indicate higher HL proficiencies. The additional response category ‘don’t know’ was recoded as ‘systematic missing’ during the analyses. Using the scoring model 0–3, the HLS-Q12 raw score sums represent a so-called sufficient statistic for the unidimensional partial credit Rasch model [46, 47]. Using a confirmatory factor analysis (CFA) and treating the data as categorical (Robust Maximum Likelihood [RML] and Diagonally Weighted Least Squares [DWLS] estimators), it was found that the HLS-Q12 data also displayed acceptable ‘goodness-of-fit’ indexes.

When applied to people with T2DM, the HLS-Q12 is sufficiently reliable with high reliability indexes (person separation index estimated to 0.847 using the RUMM2030 statistical package [48] and *Coefficient H* estimated to 0.923). All 12 items conformed well to the partial credit parameterisation of the unidimensional Rasch model, and all items had ordered response categories / no reversed Rasch-Andrich thresholds. At the overall level, the HLS-Q12 is sufficiently unidimensional (lower binominal 95% CI proportion of 0.04) with no significantly locally dependent items (cf. ‘response dependency’) and no items displaying ‘differential item functioning’ for available person factors [43].

After applying CFA using LISREL9.3 software [49], the HLS-Q12 displayed acceptable goodness-of-fit (GOF) indexes (normed Satorra-Bentler chi square of 1.67, standardised root mean square residual [SMSR] of 0.066, comparative fit index [CFI] of 0.976 and a root mean square error of approximation [RMSEA] of 0.132). The item communalities varied between 0.36 and 0.65 with an average value of 0.48 [43].

#### Health behaviour

Health behaviour was reported using single items related to physical activity, tobacco use and alcohol consumption. These items were developed based on similar items of the HLS-EU-Q47 [38]. The participants reported their physical activity by responding to the single item ‘How often have you been physically active for at least 30 minutes during the last 30 days?’, which had four response categories: none of the days (1), some of the days (2), some days every week (3) and every or almost every day (4). Respondents were classified as physically active if they reported being physically active ‘every or almost every day’. The respondents reported their tobacco use by answering the single item ‘How would you best describe your use of tobacco (such as cigarettes, cigars and tobacco pipes)?’, which had four response categories: smoke every day (1), smoke now and then (2), former smoker (3) and never smoked (4). Those who answered that they were former smokers or had never smoked were all classified as non-smokers. Alcohol consumption was reported by responding to the single item ‘How often did you drink alcohol during the last 30 days?’ with the response categories: none of the days (1), once (2), two-three times (3), once a week (4), two-three times a week (5), four-six times a week (6) and every day (7). Those who answered ‘none of the days’ were classified as non-consumers. For all items, the response category ‘do not want to answer’ was offered and later recoded as ‘systematic missing’.

#### Empowerment-related indicators

Empowerment-related indicators were measured using four self-developed items. The items were developed based on the Diabetes Empowerment Scale [50], considering the World Health Organisation’s definition of individual empowerment [29] and the aspects of patient empowerment as described by Schulz and Nakamoto [1, 51]. The four items asked how difficult or easy it is to: 1) take control of one’s own health in daily life, 2) implement goals related to one’s own health, 3) take responsibility for one’s own physical and mental health by being physical active, eating healthily and being social and 4) participate actively in health communication with health professionals by asking questions and prompting a plan of action for one’s own health needs. The items were used as a proxy for empowerment and had a four-point response scale: very difficult (1), difficult (2), easy (3) and very easy (4), where higher scores indicated higher empowerment.

### Data analysis

When investigating HL across different levels of person factors, the raw data obtained from the HLS-Q12 were transformed into logit values using RUMM2030 statistical package [48]. Hence, the analyses were performed with person-location estimates of HL, providing continuous data and interval levels. Furthermore, the demographic variables and variables related to general health, health behaviours and empowerment-related indicators were dichotomised before being entered into the analyses. Differences in HL across person factors were analysed using independent *t*-tests.

A sequential multiple linear regression analysis was performed to further investigate the association between HL and the variables that showed significant differences in HL based on the independent *t*-tests. The general linear regression model requires a continuous outcome variable and assumes normally distributed residuals with constant variance (homoscedasticity) that are independent (residual covariances are zero) and have a mean of zero. To meet the requirement of a continuous outcome variable, the HLS-Q12 person-location estimates from the Rasch modelling were used. There are no requirements for or assumptions related to the independent variables [52].

In the regression analysis, the independent variables ‘educational level’ and ‘general health condition’ were dichotomised in the same way as for the *t*-tests, whereas the empowerment-related indicators were treated as categorical with a four-point rating scale. The independent variables were entered in three sequential steps. According to Field [52], variables that are already known to be predictors of the dependent variable should be entered first. As several studies have reported a link between HL and education level [4, 12, 53–57], this variable was introduced first in Model 1. Some studies have reported a link between HL and general health, whereas the link between HL and empowerment has been investigated in people with T2DM only to a small extent. Hence, in Model 1, ‘education level’ was entered as the independent variable. In Model 2, the independent variable ‘general health condition’ was entered in the next step along with ‘educational level’. In the final model (Model 3), the independent variables for empowerment-related indicators were added. Hence, it was possible to assess the contribution of each independent variable. Adjusted R squares were used to assess the model fit, whereas a change in an R square was used to assess the contribution of newly entered independent variables [52]. The standardised *β* coefficient with *p*-value was used to assess the unique contribution of each variable. Initial analyses indicated no violation of the assumptions of multicollinearity, normality, linearity or homoscedasticity. The significance level was set at 5%. It was also investigated whether gender and age could be possible confounders.

Using a sequential multiple regression in SPSS 24 [58], missing data were treated listwise, resulting in an effective sample size of 252–257.

## Results

In Table 2, the proportion of responses (%) in each response category of the 4-point rating scale is reported for each HLS-Q12 item. On average, 38% responded in either category 1 or 2 and perceived the task as (very) difficult.

**Table 2:** The proportion of responses (%) in each category of the 4-point rating scale for each HLS-Q12 item

Item no.	Label	Very difficult	Difficult	Easy	Very easy
	On a scale from very difficult to very easy, how easy would you say it is to:				
2	find information on treatments of illnesses that concern you?	2	25	64	10
7	understand what to do in a medical emergency?	3	38	53	5
10	judge the advantages and disadvantages of different treatment options?	6	56	35	3
14	follow the instructions on medication?	2	10	72	16
18	find information on how to manage mental health problems like stress or depression?	7	55	33	6
23	understand why you need health screenings?	0	3	68	28
28	judge if the information on health risks in the media is reliable?	7	60	30	4
30	decide how you can protect yourself from illness based on advice from family and friends?	7	52	37	4
32	find information on healthy activities, such as exercise, healthy food and nutrition?	2	13	70	16
38	understand information on food packaging?	10	43	40	7
43	judge which everyday behaviour is related to your health?	1	13	70	16
44	make decisions to improve your health?	2	35	51	11
average		4 (49/12)	34 (403/12)	52 (623/12)	11 (126/12)

The item number corresponds to the specific item number in the European Health Literacy Survey Questionnaire (HLS-EU-Q47)

### HL and demographic variables, general health, diabetes outcome, health behaviours and empowerment-related indicators

Based on the investigation of HL across the levels of person factors and general health using independent *t*-tests, those with an education level at the university/university college level and those who reported their general health as good had significantly higher HL (person-location estimates of HL) with *p*-values of 0.001 and 0.002, respectively. No significant differences were observed for the person factors gender, age or HbA1c (Table 3). In addition, there were no significant differences in HL observed across age groups when treating the variable age as categorical (not shown in the table). When investigating HL between those who reported (*n* = 312) their latest HbA1c against those who did not (*n* = 76), it was found that the former group had significantly higher HL (*p* = 0.044; not shown in the table). No significant differences in mean values were observed for the health behaviour variables, but those who reported the empowerment-related indicators as easy had significantly higher HL (*p* < 0.001 for all four items).

**Table 3:** Health literacy across levels of person factors, general health, diabetes outcome, health behaviours and empowerment

	*n* (%) / mean (sd)^a^	HL mean (sd)	*P* value
Gender	*n* (%)		
Male	207 (53)	0.653 (1.43)	0.149
Female	165 (43)	0.881 (1.62)	
Education	*n* (%)		
Compulsory comprehensive/upper secondary school	196 (51)	0.599 (1.50)	0.001
University/university college	118 (30)	1.195 (1.70)	
Age			
≤ 73 years, *n* (%)	187 (48)	0.748 (1.47)	0.922
≥ 74 years, *n* (%)	188 (48)	0.763 (1.57)	
General health	*n* (%)		
Very good/good/fairly good	315 (82)	0.892 (1.55)	0.002
Bad/ very bad	64 (17)	0.235 (1.34)	
HbA1c (%)^b^			
≤ 7.0%, *n* (%)	132 (34)	0.953 (1.51)	0.319
≥ 7.1%, *n* (%)	180 (46)	0.778 (1.54)	
Health behaviour			
Physical activity^c^	*n* (%)		
Every or almost every day	119 (31)	0.772 (1.46)	0.997
Less activity	235 (61)	0.772 (1.52)	
Health condition not compatible with physical activity	17 (4)		
Missing	17 (4)		
Smoking^d^			
Daily or now and then	16 (4)	1.491 (1.42)	0.052
Never or former smoker	361 (93)	0.733 (1.52)	
Missing	11 (3)		
Alcohol consumption^e^			
No	177 (46)	0.740 (1.58)	0.728
Yes	195 (50)	0.795 (1.47)	
Missing	16 (4)		
Empowerment-related indicators			
Control of own health	*n* (%)		
Difficult	120 (31)	0.229 (1.53)	< 0.001
Easy	251 (65)	1.044 (1.46)	
Missing	17 (4)		
Implement goals about own health			
Difficult	204 (53)	0.425 (1.32)	< 0.001
Easy	166 (43)	1.213 (1.63)	
Missing	18 (5)		
Take responsibility for own health			
Difficult	127 (33)	0.213 (1.32)	< 0.001
Easy	243 (63)	1.075 (1.54)	
Missing	18 (5)		
Participate actively in health communication			
Difficult	111 (29)	0.086 (1.20)	< 0.001
Easy	223 (57)	1.105 (1.61)	
Missing	54 (14)		

HL: person-location estimates (logit values) of health literacy by means of the HLS-Q12 of Finbråten et al. [41]. Higher values indicate higher HL

^a^Continuous data: mean (standard deviation [sd]); categorical data: frequencies, n (percentage [%])

^b^Highest recommended HbA1c level in people with T2DM is 7% (53 mmol/mol)

^c^How often have you been physically active for at least 30 min during the last 30 days?

^d^How would you best describe your use of tobacco?

^e^How often did you drink alcohol during the last 30 days?

Those who reported that they had been in contact with a nurse with regard to their health situation during the last year (*n* = 214) had significantly higher HL (*p* = 0.021) than those who had not (*n* = 150; not reported in Table 3).

### The association between HL and education, general health and empowerment-related indicators

After entering the variables that showed significant differences in HL proficiency (education, self-reported general health and empowerment-related indicators) as independent variables in a sequential multiple linear regression analysis, the variables of the final model (Model 3) explained 33% of the total variance in HL as the dependent variable. The empowerment-related indicators explained more of the observed variance in HL than the other independent variables (change in R square = 28%; Table 4). The items ‘take control of one’s own health in daily life’ and ‘participate actively in health communication with health professionals’ made the strongest and most significant contributions to the variance in HL in the final model (*β* = 0.23, *p* = 0.001 and *β* = 0.28, *p* < 0.001, respectively), followed by the variable ‘education’ (*β* = 0.13, *p* = 0.015; Table 4).

**Table 4:** Regression analysis for education, self-reported general health and empowerment-related indicators on health literacy

	R square	Adjusted R square	R square change	*p*-value	Standardised *β* coefficient	*p*-value
Model 1^a^	0.030	0.026	0.030	0.005		
- education					0.173	0.005
Model 2^b^	0.067	0.060	0.037	0.002		
- education					0.156	0.011
- general health					0.194	0.002
Model 3^c^	0.346	0.330	0.278	< 0.001		
- education					0.128	0.015
- general health					0.027	0.625
- empowerment						
• control					0.233	0.001
• implement goals					0.127	0.079
• responsibility					0.090	0.194
• participate actively					0.284	< 0.001

*Note.* This table reports results from the sequential multiple regression analysis with health literacy as the dependent variable. The independent variables ‘education’, ‘general health’ and empowerment-related indicators were entered in the analysis in three sequential steps. Statistical significance was assumed at *p* < 0.05

^a^*n* = 257

^b^*n* = 256

^c^*n* = 252

Despite the significant differences in HL between those who reported that they had been in contact with a nurse with regard to their health situation and those who had not, this variable did not make a significant contribution in the regression analysis (not shown in the table). The variables gender and age did not correlate with the dependent variable, nor display any significant contribution to the models (not shown in the table).

## Discussion

On average, 38% reported that the HL tasks were difficult or very difficult. When measuring HL by means of the HLS-Q12, higher HL was associated with education at the university/university college level, good general health and higher empowerment.

### HL and demographic variables, general health, diabetes outcome, health behaviours and empowerment-related indicators

According to the proportion of responses in each response category, over one-third marked the HL tasks as difficult or very difficult, which implies that health professionals should pay attention to the individual’s HL and should adapt their health communication to the appropriate level. In contrast to van der Heide [4] and Hussein, Almajran and Albatineh [53] but in accord with Al Sayah et al. [16] and Vandenbosch et al. [54], no significant differences in HL with regard to age were found. However, the average age of the sample was relatively high, so differences related to age may not have been evident.

Significant differences in HL were found in relation to education. People with T2DM who had completed a university-level education reported a significantly higher HL (as measured by the HLS-Q12) than those with a lower education level, which is similar to the findings of previous studies [4, 12, 53–57]. This finding was also supported when using the FCCHL to measure HL in the same population [11]. According to this finding, nurses and other health professionals should tailor health information to the individual’s HL level and their educational level, and they should be prepared to devote more time to explaining relevant health information, using different learning aids and ensuring that the individual thoroughly understands the information. Health professionals should also ensure that individuals have the capacity to use the information to promote their health in everyday life. Regarding the significant association between HL and education, actions aimed at strengthening HL should be implemented as early as primary and secondary school. Such interventions should aim to strengthen reading and writing skills, strengthen knowledge related to health issues and the determinants of health, strengthen competence in the critical assessment of different sources of health information and strengthen knowledge regarding where to find evidence-based health information.

Those with a higher HL reported significantly better general health than those with lower HL estimates. However, only 4% of the variance in HL was explained by general health condition. Whether low HL could result in worse health or whether the opposite occurs is unclear. If low HL causes worse health, adapting health information to the individual’s HL is important to avoid a negative impact on health. Strengthening HL could improve an individual’s health, and thus a particular effort should be made to develop HL in people with T2DM. This is also supported by Al Sayah et al. [12], who called for studies investigating whether such interventions could lead to improvements in health.

It was also found that those who had been in contact with a registered nurse regarding their health situations had significantly higher HL. In Norway, people with T2DM are usually offered a follow-up by their regular general practitioners [45]. Those who are offered additional follow-ups with registered nurses may receive more comprehensive information and information better adapted to their needs. Registered nurses may conduct longer consultations than general practitioners due to time constraints, and thus they have time to adapt and to explain the information more thoroughly. This is potentially supported by Tshiananga [59], who found that nurse-led follow-ups and diabetes education were associated with improved glycaemic control. Thus, it could be recommended that people with T2DM should be offered follow-ups with registered nurses who are educated in diabetes treatment and health communication in addition to the follow-ups provided by general practitioner. However, many nurses have limited knowledge related to HL [60, 61], which could affect the learning outcomes of diabetes education and self-management in people with T2DM. Hence, nursing education should include a greater emphasis on HL as well as how health communication could be better adapted to people’s HL levels.

No significant differences were found in HL related to HbA1c, which is in accord with Lee et al. [57], Al Sayah et al. [16] and Finbråten et al. [11] (the latter using the FCCHL in the same population) but in contrast to van der Heide et al. [4], Powell et al. [5], Schillinger et al. [7], Tang et al. [8] and Cavanaugh et al. [9]. However, significant differences were found in HL between those reporting and those not reporting their latest HbA1c levels. The reason for people not reporting this value is uncertain. One reason could be that they do not know their latest HbA1c levels. Hence, it is possible that there is an association between HL and knowing the HbA1c level. The findings confirm the claim put forward by Al Sayah et al. [13] and Bailey et al. [14] that the association between HL and HbA1c is inconsistent. This inconsistency may be due to HL being measured using different instruments. All the studies mentioned [4, 5, 7–9, 16] with the exception of Lee et al. [57] and Finbråten et al. [11] measured HL using instruments that were limited to the functional HL level. Another explanation might be different operationalisations of the variable HbA1c. When treating HbA1c as a dichotomous variable, van der Heide et al. [4] did not find a significant difference in HL.

Despite the association between HL and self-reported general health, there was no association between HL and health behaviours. The absence of an association between HL and both smoking behaviours and alcohol consumption is in line with the findings of Friis et al. [21]. However, the association between HL and physical activity, as described by Friis et al. [21] and van der Heide et al. [4], was not supported by this study. Measuring HL using the FCCHL, Shin and Lee [62] did not find any significant direct effect of HL on physical activity in elderly people with diabetes. The average age in the sample was quite high. Hence, T2DM in the sample could be linked more to age than to health behaviours. The association between HL and health behaviours should be further explored in younger individuals with T2DM. Since the study was initiated, the recommendation for physical activity has changed from 30 min of physical activity per day to 150 min of physical activity each week [63]. However, dividing 150 min by 5 days equals 30 min 5 days per week. As this change can be considered quite small, it is not expected to influence the results of this study.

Theoretically, HL has been linked to empowerment [2, 32]. A significant association between HL and all four empowerment-related indicators was found. A relationship between personal empowerment and HL was also identified by Wang et al. [27]. The empowerment-related indicators also contributed the most to the variance in HL when performing the regression analysis. If Nutbeam’s theory [32] is assumed to be correct and thus higher HL implies higher empowerment, strengthening HL might lead to greater empowerment. Hence, to enhance empowerment in people with T2DM, nurses and other health professionals should strengthen their HL, such as by providing valid and reliable health information and guiding the individuals to the actual sources of the information. Furthermore, nurses and other health professionals should guide individuals in evaluating the advantages and disadvantages of the different treatment options and should facilitate their active participation in health communication. In addition, they should enable individuals to take control of their own health in their daily lives by providing adapted health information. The association between HL and taking control of one’s own health could also confirm the theoretical association between HL and empowerment, as ‘control’ is a central part of the definition of individual empowerment. However, higher HL does not necessarily entail empowerment [1]. Schulz and Nakamoto [1] pointed out that mismatches of these two factors can have dangerous consequences, as emphasising a high degree of empowerment without adequate knowledge and a high level of HL may increase the risk of making inexpedient health choices.

By defining empowerment as a process, Lee et al. [57] claimed that there is a direct pathway from empowerment to HL. Hence, if the individual’s active participation and involvement in health communication is facilitated, it may also strengthen HL. It is important that nurses and other health professionals facilitate this involvement by using language that is adapted to the patient’s HL level and framework of understanding. This is in accord with Engström, Leksell, Johansson and Gudbjörnsdottir [64], who found that some health information was difficult to understand due to the use of jargon and that people wanted the information to be tailored to their needs. Engström et al. [64] also found that most people with diabetes (both Types 1 and 2) wanted to participate in shared decision making. Hence, nurses and other health professionals should ask the patients about their preferences and should invite them to participate in a dialogue regarding their health and the requirements associated with living with T2DM.

The final regression model explained 33% of the variance, which means that other factors also contributed to the variance in HL. In light of the definition provided by Sørensen et al. [2], it is reasonable to assume that factors such as motivation, diabetes knowledge and general literacy may contribute to the variance in HL. Additional factors, such as self-efficacy, may also contribute to the variance in HL. There may also be person factors other than those mentioned that contribute to the variance.

#### Methodological considerations

The sample was drawn from the member list of the NDA. Hence, the sample might not be representative of the entire population of people with T2DM, as members of such an organisation may be better informed and motivated in managing their chronic disease than non-members. However, it was difficult to recruit non-members as we were not allowed to use Norwegian Patient Registry as a sampling frame. In addition, the mean age of the sample was quite high and was higher than the average age in the member list. Consequently, the results may be considered a reflection of older adults with T2DM.

We do not have access to information about age and educational level in Norwegians with T2DM not being members of the NDA. However, in a study describing the incidence of T2DM in Norway [44] from 2009 to 2014, people aged 55 and older constituted 75% of all individuals newly diagnosed with T2DM. Ruiz et al. [44] reported that about the half of the individuals newly diagnosed with T2DM had completed education at upper secondary school-level, which is similar to our study. Further, Ruiz et al. [44] reported that just below one fifth had completed higher education, which is however in contrast with our sample, where 30% had such education.

Despite the high average age in our study sample, the majority of the respondents considered their health as good or even very good. However, in Norway there is not unusual that elderly people rate their health as good. In fact, according to Statistics Norway [65], 60% of Norwegians 80 years old rate their health as good or very good. The associations between HL and general health, health behaviours and empowerment should be further explored in younger people with T2DM. According to Statistics Norway [66], 11% of the entire population includes daily smokers. Hence, there is a risk of a response bias in the study because only 4% reported being a smoker.

Responding to self-administered measures could be quite challenging for elderly people and for those with limited HL, as it requires reading and reading comprehension abilities. However, during cognitive interviews, the participants reported that the items were clearly stated and easy to understand. Advantages of using paper- and-pencil questionnaire are that there are lower risk for interviewer bias and that the respondents answering according to what they think is expected [67].

The sample size was rather small, which might lower the generalisability of the findings. However, assuming 80% power, the number of independent variables and a small to medium effect size, the sample size could be considered sufficient [52, 68].

To ensure the plausibility of the interpretations of the scores obtained from the HLS-Q12 for people with T2DM, the psychometric properties of the scale were assessed. Applying the Rasch model analysis and CFA, the HLS-Q12 was found to have acceptable psychometric properties for people with T2DM. These findings are similar to those of Finbråten et al. [42], who validated the HLS-Q12 in the Norwegian population. However, in contrast to Finbråten et al. [42], a discrepancy between the fit indexes CFI and RMSEA was found, which suggested a good and a relatively bad fit, respectively. This discrepancy was further explored by comparing estimates obtained from ML and DWLS, which displayed quite similar results. Moreover, the LISREL output did not suggest any modification indices. However, Brown [69] claimed that a high value of RMSEA may be of less concern if other GOF indexes suggest acceptable model fit. Altogether, the HLS-Q12 showed several strengths, as the data displayed a good fit to the unidimensional Rasch model, which implies that all requirements of the fundamental measurement were met [70]. Hence, the HLS-Q12 could be used as a valid and reliable scale for measuring HL in people with T2DM in relation to different health settings. However, the GOF indexes should be further explored in future studies. The HLS-Q12 has thus far been validated for people with T2DM and in the Norwegian population, but the psychometric properties of the instrument should be further assessed in other populations and across different languages and cultures.

## Conclusions

When applying the HLS-Q12 to measure HL in people with T2DM, it was found that a higher HL was associated with education at the university/university college level, good self-reported general health and higher empowerment-related indicators. Nurses and other health professionals should be aware that people have different levels of HL and should pay particular attention to the prerequisites for dealing with health information among those with less education and worse self-reported health. Furthermore, the health information should be adapted to the individual’s HL to support the individual’s active participation in diabetes management and to enable individuals to take control of their own health in their daily lives. No evidence of an association between HL and glycaemic control nor between HL and health behaviours was found.

The association between HL and health behaviours should be further investigated, especially in younger people with T2DM. Other factors that could contribute to the variance in HL should also be investigated.

## Data Availability

The dataset used and analysed during the current study is available from the corresponding author upon reasonable request.

## Abbreviations

CFA: Confirmatory factor analysis
CFI: Comparative fit index
CI: Confidence interval
DWLS: Diagonally Weighted Least Squares
FCCHL: Functional, Communicative, and Critical Health Literacy scale
GOF: Goodness-of-fit
HbA1c: Glycated haemoglobin
HL: Health literacy
HLS-EU-Q47: European Health Literacy Survey Questionnaire
ML: Maximum Likelihood
NDA: Norwegian Diabetes Association
RMSEA: Root-mean-squared error of approximation
SRMR: Standardised root mean square residual
T2DM: Type 2 diabetes
